# Recent advances in biodegradable matrices for active ingredient release in crop protection: Towards attaining sustainability in agriculture

**DOI:** 10.1016/j.cocis.2020.05.002

**Published:** 2020-08

**Authors:** Tahira Pirzada, Barbara V. de Farias, Reny Mathew, Richard H. Guenther, Medwick V. Byrd, Tim L. Sit, Lokendra Pal, Charles H. Opperman, Saad A. Khan

**Affiliations:** 1 Department of Chemical & Biomolecular Engineering, North Carolina State University, Raleigh, NC, USA; 2 Department of Entomology & Plant Pathology, North Carolina State University, Raleigh, NC, USA; 3 Department of Forest Biomaterials, North Carolina State University, Raleigh, NC, USA

**Keywords:** Biodegradable matrix, Crop protection, Active ingredient release, Agriculture, Formulation, Seed coating, Encapsulation, Electrospinning, Nanofibers

## Abstract

Climate changes, emerging species of plant pests, and deficits of clean water and arable land have made availability of food to the ever-increasing global population a challenge. Excessive use of synthetic pesticides to meet ever-increasing production needs has resulted in development of resistance in pest populations, as well as significant ecotoxicity, which has directly and indirectly impacted all life-forms on earth. To meet the goal of providing safe, sufficient, and high-quality food globally with minimal environmental impact, one strategy is to focus on targeted delivery of pesticides using eco-friendly and biodegradable carriers that are derived from naturally available materials. Herein, we discuss some of the recent approaches to use biodegradable matrices in crop protection, while exploring their design and efficiency. We summarize by discussing associated challenges with the existing approaches and future trends that can lead the world to more sustainable agricultural practices.

## Highlights

• Providing food safety and security is critical for the growing global population.
• Crop yield is affected by various biotic and abiotic factors.
• Targeted/sustained delivery of agrochemicals reduces excessive use of pesticides.
• Nature-derived biodegradable materials curtail plant health and environmental harm.
• Biodegradable matrices hold promise for sustainable crop protection.

## Graphical abstract

**Figure:**
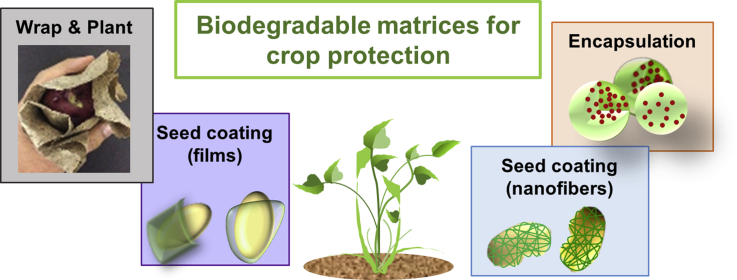

## Introduction

The ability to provide sufficient, safe, and nutritious food to the global population, which is estimated to reach 10 billion by 2050 [1,2], is becoming a major challenge for agriculture, especially because of various biotic and abiotic stresses impacting crop yield and quality. The Food and Agriculture Organization of the United Nations (FAO) estimates worldwide economic losses of approximately $220 billion annually owing to plant diseases, while damage caused by plant pests results in about a 20–40% loss in the total crop production globally [3]. Evolving/emerging species of plant pathogens and pests, climate changes, and limited availability of clean water and arable land have exacerbated the challenge [3]. To meet the United Nations' Sustainable Development Goals of ending hunger and improving human well-being with minimum environmental impact, there is a dire need for the development of sustainable agricultural production systems, both in mechanized large-scale production systems and smallholder farms in the developing world [4]. Therefore, agricultural production systems in the 21st century must aim to embrace technological innovations to provide sustainable solutions for both production and environmental stewardship to provide better global food security and adhere to Sustainable Development Goals [4,5].

**Figure 1:**
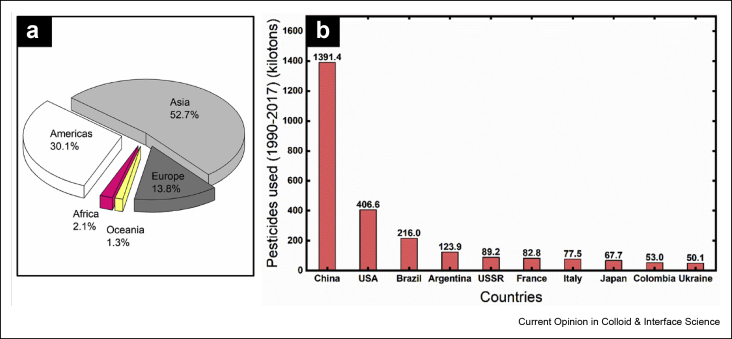
Global pesticide consumption from 1990 to 2017. **(a)** Percentage of pesticides used in Africa, Americas, Asia, Europe, and Oceania. **(b)** Total amount of pesticides used by top ten pesticide-consuming countries (data replotted from FAO [7]). FAO, Food and Agriculture Organization of the United Nations.

The increased demand for food for an exponentially growing global population in the 20th century led to various approaches for increasing and improving crop yield, including deployment of genetically modified crops and increased application of agricultural chemicals and fertilizers. However, the generation of better quality and high-yielding cultivars has resulted in the evolution of novel genotypes of pests that evade or overcome inherent host defenses or are resistant to exogenously applied pesticides [6]. While the concept of crop protection is as old as the history of agriculture, most of the earliest approaches focused on natural methods and agents such as mulching, burning, or use of natural oils and beneficial insects. During the past century, the application of pesticides has developed as an important management strategy to combat plant pests and pathogens. The advent of chemical pesticides that occurred after World War II led to these synthetic compounds becoming a widely accepted management strategy for controlling plant pests and pathogens in resource-rich and resource-poor nations alike. Figure 1a depicts the average use (percentage) of pesticides consumed across the globe from the year 1990 to 2017. Asia represents the major consumer, with the Americas coming in second. Among the top ten pesticide-consuming countries, China used more than 50% of the total pesticides consumed by all other countries during that period (Figure 1b) [7].

**Figure 2:**
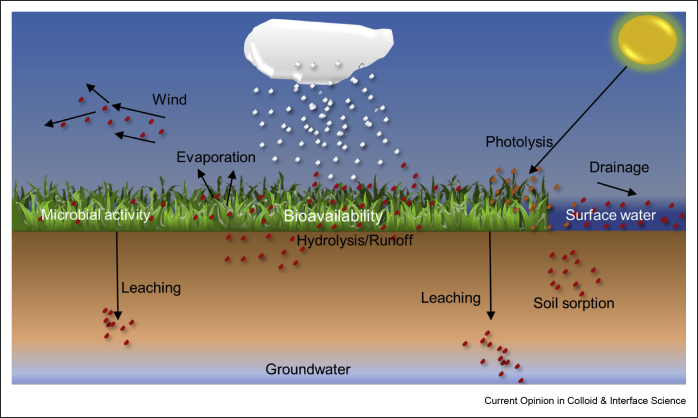
Pathways of pesticides after spraying. While a fraction of it is bioavailable to the target organisms, most of the sprayed pesticide is lost because of photolysis, hydrolysis, soil leaching, sorption, evaporation, drainage, and microbial activity, resulting in nontargeted damage to the environment and health.

The FAO reports an increase of 1.09 kg per hectare of cropland in global pesticide use from 1990 to 2017 [7]. As the sale of pesticides increased around the world, their excessive use resulted in soil, air, and water contamination, which in turn caused the death of numerous nontarget organisms, including bees, small mammals, birds, and fish [8]. About twenty-five million agriculture workers suffer from pesticide poisoning each year owing to excessive exposure [9]. It is also well established that during conventional applications, more than 90% of the pesticides are wasted because of photodegradation, wind, hydrolysis, evaporation, drainage of surface water, leaching into the soil, and microbial activity, leaving behind a small fraction to be biologically available for the target organisms (Figure 2). Indiscriminate use of pesticides has also culminated into what growers refer to as the ‘pesticide treadmill,’ a metaphorical loop in which increased pesticide use leads to the development of resistance in pests [8]. Because it is unlikely that the demand for pesticides will subside anytime in the near future, rising environmental concerns and health risks because of excessive use of pesticides have led to attempts to develop alternative approaches for minimized and targeted use of pesticides [10]. Controlled and targeted release of a pesticide can assist in increasing its bioavailability for prolonged and sustained crop protection, ultimately leading to minimizing development of pest resistance, reduced pesticide residues in food and field, and reduced air pollution.

Different approaches adopted to overcome the negative impacts of overuse of pesticides include precision agriculture [11], biopesticides [6,12], encapsulating agents [13], and seed coatings [14]. Precision agriculture deals with the utilization of various in-field, near-range, and remote sensing techniques to identify problem areas that may need timely intervention [11], whereas biopesticides are naturally occurring substances that control pests via predatory, parasitic, or chemical relationships [6]. Although important to the future of sustainable agricultural production systems, a detailed discussion of these topics falls outside the realms of this review. Our focus lies in alternate biodegradable carriers, derived from natural sources that are being developed to deliver ultralow volumes (ULVs) of agricultural chemicals. While encapsulation of an agrochemical in a suitable carrier results in its targeted and controlled delivery with enhanced adhesion to the substrate, the polymer matrix also protects the loaded cargo from exposure to the external environment, preventing its evaporation and degradation. The combination of an ULV of agrochemicals loaded on biodegradable carriers would reduce the deleterious impact of synthetic pesticides and carriers on crops and the environment. For brevity, we focus on some of the current and emerging sustainable technologies. Starting with a brief background of traditional methods used for crop protection, we discuss various approaches for seed coating, ranging from industry-established methods to more recently explored techniques such as electrospinning and smart delivery of pesticides and/or nutrients via encapsulation. We conclude with a deliberation on a recently developed, simple, and cost-effective ‘Wrap and Plant’ (W&P) technology.

## Cultural approaches

Traditional techniques, referred to as ‘cultural methods,’ are widely used across the globe to reduce plant pest and pathogen populations without the application of synthetic chemicals. Some of these approaches include crop rotation, creating a fallow (noncrop) period, or flooding the fields for a specific period to create anaerobic conditions [15]. However, each of these techniques presents a unique challenge. For instance, rotating with nonhost crops may be economically difficult for farmers. In addition, although a given crop might not be a host of the target pest/pathogen, another species could potentially fill the niche. For example, for protection against the burrowing plant parasitic nematode (PPN), bananas are often rotated with sweet potatoes. However, sweet potatoes are excellent hosts for another PPN, the guava root-knot nematode, *Meloidogyne enterolobii* [16]*.* Management strategies that include creating a fallow period or flooding the fields may also not be an economical alternative, especially in resource-poor countries [16].

Another deployed management strategy includes releasing biological control agents such as bacterial or fungal spores that are antagonistic or parasitic to the pest/pathogen of interest. Several strains of saprophytic fungi of the genus *Trichoderma* (*T*.) have been used as biocontrol agents against numerous plant pathogens [17]. For instance, isolates of *Trichoderma harzianum* and *T. viride* have been effective in reducing populations of the PPN *Meloidogyne javanica* on tomatoes under greenhouse conditions [18]. However, these experiments were conducted under controlled conditions, and the results cannot be reliably applied to field conditions, in which factors such as the inherent microbiome of the soil may have an impact on the potency of the biocontrol agent. Furthermore, experiments with other biocontrol agents such as *Fusarium oxysporum* strain 162 and *Bacillus firmus* demonstrated reasonable control against the phytoparasitic nematode, *Radopholus similis.* Much like synthetic chemicals, these agents are expensive to apply, and the protection achieved is restricted to one growing season, possibly owing to viability issues [19].

## Biodegradable seed coatings

**Figure 3:**
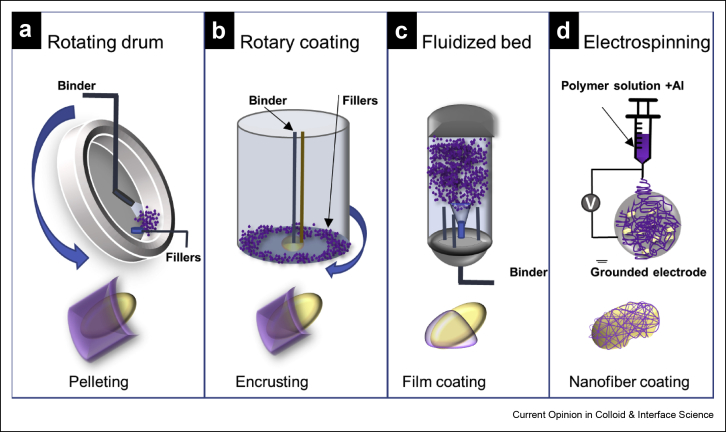
Schematic of various seed coating approaches (partially adapted from the studies by Farias et al. [14] and by Pedrini et al. the study by Pedrini et al. [21], Copyright American Chemical Society, 2019 and Elsevier, 2017). Blue arrows represent the direction of the moving parts of the setup. **(a)** Rotating drums depend on a slow rotating motion and gradual addition of materials to increase the pellet size. The liquids are applied using a spray nozzle, and fillers are added via a hopper or manually on the seeds placed in the pan. **(b)** Rotary coating allows for both film coating and pelleting. **(c)** Fluidized bed is used for film coating on the seeds that are subjected to subfloor air flow to remain air buoyant in the cylinder. All these approaches involve coating the seeds with the AIs either independently or mixed with the filler or the binder. **(d)** Nanofiber coating involves electrospinning a polymer solution loaded with the AI) directly on the seeds under the force of an electric field. AI, active ingredient.

In contemporary agriculture, seeds are often treated with agrochemicals, such as fungicides, pesticides, or fertilizers, to protect them from pathogens and insects, ensuring crop establishment and maximum yield [20]. Seed treatment is generally considered a robust approach when compared with traditional soil amendments because of the targeted and controlled delivery of active ingredients (AIs), which minimizes their overuse and decomposition. Over the years, seed coating has become widely used in agriculture and horticulture sectors worldwide, with a value of US $53.76 billion in 2014. With the introduction of this technology in developing countries, the global market of seed-based coating is expected to reach US$ 1.63 billion by the year 2020 [21]. Seed coating is a practice that involves the application of exogenous materials onto a seed surface to modify its properties and/or to deliver AIs without affecting its size and shape [21,22]. Currently, the standard in the industry is to use a film for seed coating, which is usually obtained by the application of a polymer suspension via a fluidized bed or a rotary coater [20, 21, 22]. In the past decade, biopolymers, such as hydroxyethylcellulose [23], chitosan (CS) [24], gelatin–gum arabic [25], and, more recently, a mixture of biopolymers [26], have been used as substitutes to synthetic materials owing to their environment friendly and nontoxic characteristics, as well as good adherence to the seeds [21,26]. Figure 3 displays a schematic representing various seed coating approaches that we will be discussing in the subsequent sections, and Table 1 summarizes some of the recent strategies explored to establish sustainable crop protection using biodegradable matrices derived from natural sources.

**Table 1:** Crop protection approaches exploring biodegradable matrices derived from natural resources.

Category	Carrier		Fabrication process	Special features
	Composition	Physical form		
Seed coatings	Starch, gelatin, and PVA [26] Starch-based bioplastic [20] Cellulose diacetate [14]	Film Film Nanofibers	Filmogenic solutions Rotating drum coating Electrospinning	Low-cost coatings with controlled water solubility and a release profile High adhesion to the seed coat, resulting in reduced dust release Slow and sustained release of the AI with maintenance of coating integrity in water
Encapsulation	Chitosan and cyclodextrin [41] Chitosan–cinnamic acid [42] Sodium alginate and calcium chloride [43] Starch–alginate–clay–rice husk [44] Alginate and lignin [45] Lignin [46] Lignin [47] Cellulose acetate [48] Nanofibrillated cellulose, starch, and urea [49] Microcrystalline cellulose and lignin [49]	Nanoparticles Nanogel Nanoparticles Microsphere Microparticles Nanoparticles Microspheres Beads Nanocomposite granules Hydrogels	Kneading Precipitation Emulsion cross-linking Cross-linking and ionic gelation Microfluidics Nanoprecipitation Organosolv and ionic isolation Supercritical impregnation Extraction, lyophilization, cross-linking Freezing and chemical cross-linking	Amphiphilic, not studied on crops but on mites No negative impact on organoleptic properties of maize Controlled release profile of AIs Composition-selective and sustained release of AIs Enhanced phytotoxicity and better controlled release profile UV protectant and antioxidant Increase in dosing time and reduced leaching of the AI Enhanced release of the AI in water Water uptake–induced diffusion and barrier effects Composition-dependent release and swelling
Seed treatments	Lignocellulosic matrices [51]	Seed wraps	Solvent-free manufacture from waste banana fibers	Cost-effective and sustainable with no toxic chemicals or complicated processing

AI, active ingredient; PVA, polyvinyl alcohol.

### Film coating

**Figure 4:**
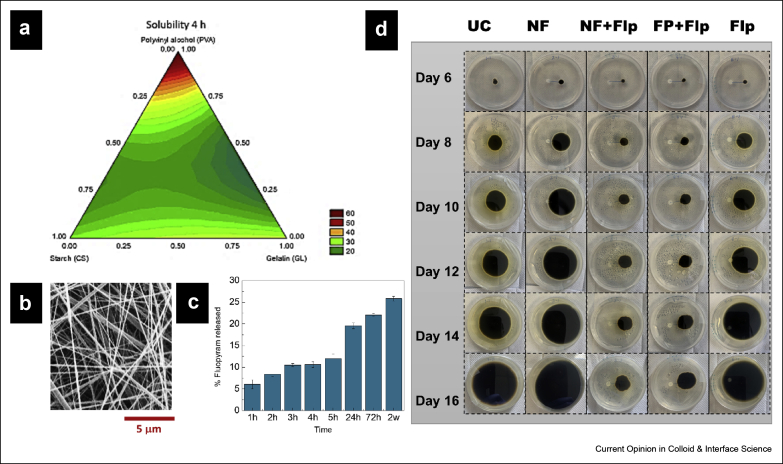
AI loaded film and electrospun nanofiber based seed coatings. **(a)** Contour plot displaying solubility (after 24 h) of films developed from gelatin, PVA, and starch. There is a rise in solubility with increasing PVA and decreasing starch content. Reprinted with permission from Vercelheze et al. [26], Copyright Springer 2019. **(b)** SEM image showing CDA nanofibers containing fluopyram as the AI. **(c)** Release kinetics of fluopyram from CDA nanofibers measured via HPLC. **(d)** Growth of *Alternaria lineariae* assay over time for fluopyram-loaded nanofibers (NF + Flp) versus controls (untreated [UC], commercial version of fluopyram [Flp], fluopyram-loaded filter paper [FP + Flp], and nanofibers [NFs] only). Reprinted with permission from Farias et al. [14], Copyright American Chemical Society 2019. CDA, cellulose diacetate; AI, active ingredient; HPLC, high-performance liquid chromatography; PVA, polyvinyl alcohol; SEM, scanning electron microscopy.

Seed coatings are primarily divided into three categories, film coating, encrusting, and pelleting, depending on the weight and size of the coat (Figure 3a–c). Film coating entails applying a thin (less than 10% of seed weight) coat on the seed, whereas encrusting and pelleting result in comparatively thicker coats, approximately ∼100–500% and >500% of seed weight for encrusting and pelleting, respectively [21] (bottom parts of Figure 3a–c). The thick coating during pelleting makes it difficult to distinguish the initial seed shape [21]. Apart from the AI (protectant, tracer, colorant, nutrient, symbiont, soil adjuvant, and phytoactive promoters), a typical seed coating mixture is composed of binders and fillers [21], in which the binders are polymeric materials (both natural and synthetic) in a liquid state that stick to the seed surface and carry AIs. Fillers are mostly inert powdered substances, such as talc, sand, or clays, used to increase seed shape and size. Both binders and fillers need to be compatible with the AIs. It has been recently reported that coating the seeds helps to enhance the plant stress tolerance during seed germination and seedling growth [27]. In an attempt to reduce the fossil fuel–based polymers and owing to the biodegradable nature, ease of availability, and generally low cost of the precursors, biodegradable polymers have been used in seed coating over the last two decades. Gelatin [25], gums [25,28], CS [24,29], cellulose and its derivatives [23,29,30], starch [26,31], and alginates [32] are some of the biopolymers that are used alone or in combination with other materials as seed coats for targeted delivery of AIs and enhanced growth and preservation of plants. However, most of the biopolymers have to be used with additional substances, that is, surfactants and film formers. Ren et al. [27] have developed a polyacrylamide and carboxymethylcellulose–based seed coat for wheat seeds using various fungicides and additives to investigate the effect of seed coating on germination and growth of wheat. To improve the seed coating efficiency, Berninger et al. [32] have recently fabricated a technique to control the size of alginate beads for improved seed coating of maize seeds to ensure sustained delivery of plant growth–promoting bacteria. However, one of the major drawbacks of biopolymers, especially polysaccharide-based seed coats, is the hydrophilic nature of the seed coat [33]. More recent research is focused on the development of hybrid coats that display reduced water solubility to maintain the integrity of seed coats for prolonged periods. Vercelheze et al. [26] studied the efficacy of a hybrid coat of polyvinyl alcohol (PVA)–starch and gelatin on maize seeds and noticed improved viability of loaded *Azospirillum brasilense* Ab-V5 for longer periods. Their studies demonstrated that both lower molecular weight and degree of hydrolysis of PVA contributed to the increase in the solubility of the coat. On the other hand, most of the abundantly available hydroxyl groups in starch were involved in intramolecular bonding between the polymer chains instead of developing hydrogen bonds with water, resulting in very low solubility of starch as compared with PVA. More interestingly, seed coating developed from mixtures of PVA and starch displays a significant reduction in water solubility, which can be attributed to the interactions between PVA and starch molecules, resulting in unavailability of hydrophilic groups to interact with water. Figure 4a, which shows a contour plot of solubility (after 24 h) of films developed from gelatin, PVA, and starch, reveals a rise in water solubility with increasing PVA and decreasing starch content. Their approach is significant for a tailored control on water solubility of seed coats by using composite films instead of single-component seed coats.

### Bioplastic–based seed coatings

Although polymer films with high adherence to seeds are already in use, the release of material due to abrasion of film-coated seeds during handling and planting is still an existing concern as it is potentially associated with bee colony mortality and other environmental issues [34,35]. In this regard, the past decade has seen a major development in the field of biobased plastics (or bioplastics), which consist of organic carbon–based molecules derived from biobased materials (plant biomass, algae, fungi) [20,34,36,37]. Being the fastest growing biobased product globally [38], bioplastics are used in various sectors including construction, automobiles, and agriculture. Although the major use of bioplastics in agriculture is in mulching, they are also considered an interesting option in seed coating [37,39].

To pursue a coating that remains adherent to the seed surface with effective reduction of dust released from the treated seeds, Accinelli et al. [20] evaluated the use of a starch-based bioplastic in a liquid formulation for film coating of corn and canola seeds with a plant growth–promoting fungus, *T. harzianum,* alone and also in combination with two synthetic pesticides. They discovered that the thermoplastic properties of starch in the bioplastic result in the generation of a thin elastic coat around the seed, with reduced abrasion and release of dust particles, as compared with the commercial coatings. Their coating also displayed no reduction in germination or seedling growth. However, it has also been observed that incorporation of insecticides in the bioplastic coating reduced their persistence in soil. More recently, the same group has analyzed the degradation of the starch-based bioplastic seed coating in the soil and noticed a faster decomposition of the starch-based seed coat than a coating with a commercial polymer [34]. Faster decomposition of bioplastic fragments contributed by the biodegradable nature of the coat accelerates the release of AIs. Furthermore, although mainly produced from biobased precursors (corn, sugarcane, and waste fats/oils), all the bioplastics are not completely biodegradable, which ultimately results in addition of unwanted materials in the soil. To address this issue, there has been a growing interest in recent years to develop bioplastics from nonfood agroforestry resources, such as lignocellulosics from wood and agricultural resources [39]. However, the complicated processing steps (*e.g.* pretreatment, detoxification of liquids, fermentation, conversion, separation, and purification) and use of expensive solvents (ionic liquids) or microemulsions for fractionation are the major drawbacks for large-scale application of bioplastic-based seed coatings. A decrease in water permeability and exchange of gases because of the film-based nature of the coatings are also considered major impediments to the approach [40].

### Electrospun nanofiber–based seed coatings

To overcome the limitations of conventional seed coatings, other approaches have been explored such as the use of laboratory mixers, seed molding [21,52], and more recently electrospun nanofibers [14,40,53, 54, 55]. Electrospinning is a technique based on the application of electrical force to produce polymer nanofibers ranging from a few nanometers to several micrometers in diameter [56,57]. In the past decades, their use has been considered for several applications, such as filtration, tissue engineering [58], and protective clothing [59]. However, their high surface area-to-volume ratio, porosity, and lack of residual solvents [40] make them desirable candidates for delivery of AIs in crop protection. More recently, experiments have been performed by various research groups to explore electrospun nanofibers for controlled delivery of AIs. Spasova et al. [60] demonstrate the use of CS-containing polyethylene oxide and polyacrylamide nanofibrous mats as a potential platform to deliver *T. viride* spores. Furthermore, the authors have shown that the viability of fungal spores and the antifungal potential of nanofibrous mats were not affected by electrospinning, thereby making nanofibrous mats a promising candidate and electrospinning a versatile platform for delivering pesticides. Electrospinning has also recently been used to fabricate microfibers/nanofibers of polycaprolactone, cellulose acetate (CA), and polyhydroxybutyrate, which are capable of sustained release of insect pheromones. These biodegradable fiber mats provided an effective platform for trapping the olive fruit fly (*Bactrocera oleae*) and olive moth (*Prays oleae*) males by controlled release of their pheromones [22]. Besides, electrospun fibers have been used to develop nonwoven breathable antifungal barriers for protection against the phytopathogen *Phaeomoniella chlamydospora*, the causative agent of esca disease in vine plants. The authors who developed the nonwoven matrix have shown that the electrospun polymer creates an effective barrier against *P. chlamydospora* on pruning wounds, while simultaneously allowing the exchanges of gases and moisture [21].

Because nonwoven nanofiber mats facilitate gas and moisture exchange when compared to traditional film coatings, Hussain et al. [54] exploited these properties to coat canola seeds with PVA and poly(vinylpyrrolidone) (PVP) composite nanofibers plasticized with glycerol containing a microbial consortium. Their study demonstrates an improved root system, plant germination, and effectiveness of the bioinoculant at the root–soil surface. However, the hydrophilic nature of the polymer blend is a major point of concern as the soil moisture can lead to fiber dissolution, accelerating the release of the AI. A similar problem was faced by Krishnamoorthy et al. [61] who coated cowpea seeds with electrospun polyvinylpyrrolidone (PVP) containing urea as the AI and compared the release profile with that of the same material cast as a film. The nanofibers showed better performance than the films in terms of coverage owing to their higher surface area, porous surface, and absence of residual solvents. Despite those advantages, pH and conductivity measurements revealed a burst release of the AI because of the hydrophilic nature of PVP. To improve coating performance, the same group developed a hybrid PVP–poly(diethoxy)phosphazene (PPZ) electrospun nanofiber coating, in which both polymers are biodegradable, and the hydrophobic nature of PPZ prevents dissolution of the coating in water [40]. However, the high cost associated with the PPZ polymer is a barrier to its application on a large scale. More recently, Farias et al. [14] successfully coated soybean seeds with electrospun cellulose diacetate nanofibers containing two different AIs, abamectin (Abm) and fluopyram (Figure 4b). They showed that the coatings did not prevent seed germination and ensured a sustained and localized release of the AIs (Figure 4c) while retaining coating integrity owing to the hydrophobic nature of the polymer. In addition, *in vitro* fungal assays showed that the fluopyram-loaded nanofibers consistently inhibited fungal growth, as shown in Figure 4d. Based on the studies discussed above, one can note that hydrophobicity of the coating plays an important role in controlling the release rate of AIs of interest. However, the current literature lacks information on whether there is an optimum level of hydrophobicity to ensure efficient delivery of AIs while avoiding germination inhibition. We hypothesize that the porosity of electrospun fibers would be enough to prevent any germination problems and that the efficient delivery of the AI will depend on the type and strength of interactions between the polymer and AI.

These studies illustrate the most recent technological advances in the area of seed coating with biodegradable materials. Although electrospun nanofibers seem to be a promising approach with many advantages when compared to the widely used film coatings, there are still several issues to be explored. These include optimization of the coating material to allow encapsulation of several AIs and the ability to coat the seeds on a large scale.

## Encapsulation of pesticides

Application of nanotechnology in agriculture is not just limited to electrospun nanofibers as controlled release matrices for AIs, rather it extends beyond pesticide delivery. For instance, nanoencapsulation of fertilizers by Kampeerapappun and Phanomkate ensured controlled release of nutrients via diffusion without degradation for 30 days [23]. Nanofibrous membranes capable of immobilizing enzymes, which could be used for detecting pesticides, have also been reported [24]. In their attempts to develop formulations that are not prematurely degradable and exhibit tunable solubility and sustained release of AIs, various research groups have developed microscale/nanoscale formulations to carry agrochemicals for targeted delivery. These formulations can easily spread on leaf and soil surfaces without impacting the natural processes, that is, transpiration, gas exchange, and photosynthesis. Furthermore, the high surface area of the carrier materials ensures targeted delivery of the AI by enhancing its release even when a low content of AI is loaded in the formulation. Although agrochemicals are reported to be incorporated in the carrier material via adsorption, entrapment, encapsulation, or attachment, nanoencapsulation is demonstrated as one of the promising techniques, with maximum efficiency and minimum damage to the environment and health [13].

Nanoencapsulation refers to the enclosure of various substances inside a secondary material, typically spherical in nature, with a diameter in the nanoscale/microscale range (1–1000 nm) [13]. There are two approaches developed to select carrier systems or encapsulating agents for pesticides. The first approach focuses on synthetic materials such as mesoporous alumina [62], polymers [13], metal–organic frameworks [63], carbon nanotubes [64], and mesoporous/hollow silica [13,65]. One of the major drawbacks while using synthetic materials is their impact on soil chemistry, which will have to suffer a new stressor in the form of nonbiodegradable nanoparticles (NPs). Recently Simonin et al. [66] discovered that the presence of titania NPs in the soil negatively affects the activity of denitrification enzymes and the inherent bacterial community in the soil. The complicated processing steps and cost of the process/precursors taken together with the nonbiodegradable nature of the encapsulating agents have led researchers to switch to a more sustainable approach focused on fabrication of biobased materials that are mostly biodegradable, for example, CS, alginates, lignin, cellulose, and starch [62].

**Figure 5:**
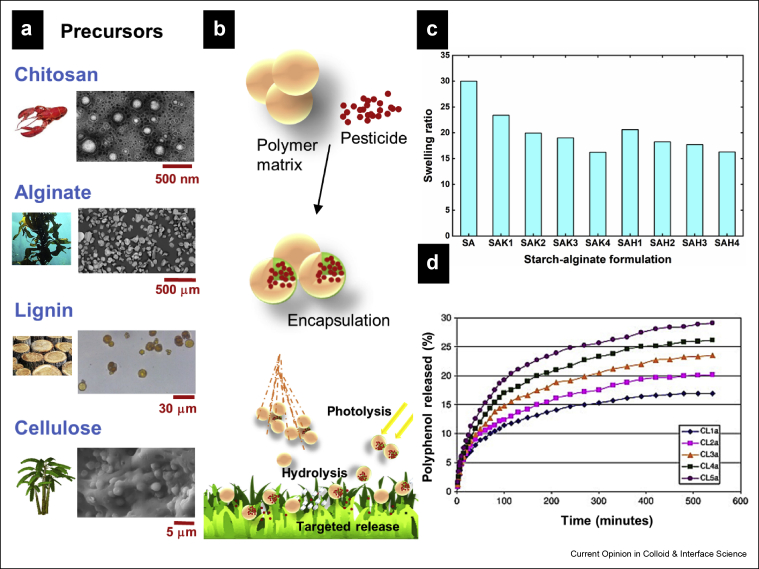
Biobased materials in AI encapsulation. **(a)** Representative biobased precursors used to encapsulate agrochemicals. SEM images showing chitosan NPs, alginate beads, organosolv lignin microparticles, and starch–cellulose nanofiber hybrid granules. Adapted with permission from Berninger et al [32], Campos et al [41], Taverna et al [47], and Patil et al [49], Copyrights Nature 2018, Taylor & Francis 2018, Elsevier 2018, American Chemical Society 2018. **(b)** Schematic of pesticide encapsulation in a polymer matrix (gels/microparticles/nanoparticles) which when sprayed on plants deliver the AI at the target with minimum losses from hydrolysis, photolysis, and runoffs. **(c)** Decrease in the swelling ratio of starch–alginate (SA) beads in PBS at 30 °C when kaolin (K) or rice husks (H) are added in different proportions. The numbers display wt. % of kaolin (K) or rice husks (H) in the bead containing a constant amount of starch (S) and alginate (A). Reproduced with permission from Feng et al. [44], Copyrights MDPI 2019. **(d)** Increasing release profiles of polyphenols with increase in lignin content from cellulose lignin hybrid (CL) hydrogels in water:ethanol medium, at 37 °C (CL1, CL2, CL3, CL4, and CL5 are the hydrogels containing cellulose [C] and lignin [L], and the corresponding numbers in the sample name indicate the ratio of lignin to cellulose). Reprinted with permission from Ciolacu et al. [50], Copyrights Elsevier 2012. PBS, phosphate-buffered saline; AI, active ingredient; NP, nanoparticle; SEM, scanning electron microscopy.

Figure 5a exhibits some of the biodegradable materials that are used to encapsulate AIs in various forms, while Figure 5b presents a schematic of the pathway of the encapsulated AI when sprayed on plants. When compared with the pathway of a nonencapsulated pesticide (Figure 2), the encapsulated pesticide shows a marked improvement for its targeted delivery, with minimal loss to the surroundings when sprayed on plants. Table 1 lists some of the approaches recently explored for the targeted delivery of agrochemicals. Commonly termed as ‘smart delivery systems/formulations’ or ‘green nanoformulations,’ these delivery systems are considered attractive options because of their biodegradability, cost-effectiveness, targeted delivery, large surface area, and thermal/UV stability [13,67,68]. Their particle size in the nanoscale range results in reduced sedimentation, leading to a stable formulation for longer periods, enhancing their scope as efficient sprayables [69].

### Chitosan

Various polysaccharides such as CS, starch, and alginates are used in pure form or in combination with other materials to encapsulate pesticides [43,44,62,65,68]. CS is a cationic linear chain polyaminosaccharide obtained from alkaline deacetylation of chitin. Its biocompatibility, biodegradability, low toxicity, and good miscibility with other polymers, as well as the chemically reactive structure, make it a suitable candidate for encapsulation of pesticides, although its highly hydrophilic nature is considered a drawback. To manipulate hydrophilicity of CS, various biobased or synthetic polymers are explored. An approach recently proposed is to cross-link it with β-cyclodextrin (β-CD) via the ionic gelation method [41]. β-CD is another polysaccharide produced from the enzymatic conversion of starch. Its hydrophilic surface and hydrophobic inner cavity make it an interesting candidate for diverse reactivity. These CS–CD NPs were used to successfully encapsulate two botanical AIs, carvacrol (a botanical insecticide) and linalool (a botanical repellent), which displayed enhanced efficiency and improved lifetimes, while the nanoformulation exhibited good colloidal characteristics. This study is significant in the field of nanoencapsulation of multiple AIs wherein a hydrophilic substance can be transported in the polymeric CS matrix, while a hydrophobic substance can be carried in the cavities of β-CD. More recently, Kujur et al. [42] and López-Velázquez et al. [70] have also encapsulated various AIs in their hybrid CS–cinnamic acid nanogel and CS–gelatin–PVA–based hydrogels, respectively. These groups observed a controlled release profile from the encapsulation as compared with the free AIs.

### Alginates

Alginates are biopolymers that have been extensively studied for their role in crop protection for decades. Their biodegradability, the presence of reactive carboxylic groups, and their ranking as GRAS (Generally Recognized As Safe) materials make them ideal candidates for sustained release of the loaded cargo. Although mostly used as gels, alginates have been recently fabricated to encapsulate AIs for targeted and controlled delivery [43,67,68,71,72]. In 2018, Patel et al. [43] successfully used alginate encapsulation for sustained delivery of cypermethrin. Using calcium chloride as the crosslinker, they observed non-Fickian diffusion of the AI from the formulation, which displayed a slower release profile than the commercial cypermethrin. Jurić et al. [71] have demonstrated controlled release of the calcium ion and a biocontrol agent, *T. viride* spores in calcium alginate microspheres. They observed a Fickian diffusion–controlled release profile of *T. viride* spores, which was inversely related to the number of calcium ions in the microsphere. Although they conducted preliminary studies to characterize the structure of the microspheres, a detailed investigation into the interactions between the components is needed to establish a release profile mechanism for developing microspheres with tailored properties. One of the major challenges associated with alginate-based carriers is the swelling of nanoparticles/microparticles because of the highly hydrophilic nature of alginates. Recently, alginates have been mixed with clays, CS, starch, or cellulose to control their release profiles [44,45]. Busatto et al. [45] fabricated alginate beads with lignin particles for controlled release of atrazine. While the lignin component of the formulation imparted improved phytotoxicity as compared with commercial atrazine, alginate helped to slow the release of atrazine as compared with lignin only. Feng et al. [44] incorporated kaolin and rice husk powder to control swelling, enhance the bioavailability of metalaxyl (AI), and slow down the release of spores of a biocontrol agent and fungicide from starch–alginate–based beads. As displayed in Figure 5c, they noticed a reduction in the swelling of starch–alginate beads when rice husks or kaolin was incorporated in the beads. They also observed a similar trend in the release profile of the AIs. Depending on the composition-dependent release kinetics of a fungal biocontrol agent and a pesticide, they suggested the suitability of starch–alginate–rice husk powder beads for use at the beginning of crop growth, whereas starch–alginate–kaolin beads were recommended to be used at later stages.

### Lignin

Lignin is the most abundant renewable aromatic biopolymer with a variable structure, depending on the plant source. Generally obtained as a by-product of the pulp and paper industry, lignin exhibits antioxidant and antimicrobial properties because of its complex structure abundant in polyphenols [62,73]. For decades, lignin has been considered an ideal protective system for UV-sensitive AIs because of its aromatic nature and antioxidant properties [62,74]. However, only recently, it has been established that nanoscale fabrication of lignin enhances its stability at extreme pH ranges and under UV light as compared with its bulk-scale counterparts [46,75]. Yearla and Padmasree [76] have prepared lignin NPs from the stem of *Leucaena leucocephala* (subabul) through nanoprecipitation. They created a prototype by encapsulating diuron in lignin NPs and conducted a detailed release study, after confirming the presence of diuron via fourier-transform infra red spectroscopy (FTIR), differential scanning calorimetry (DSC), and UV–visible spectroscopy. They observed that the encapsulation of diuron in lignin NPs results in slow and sustained release of diuron at a wide range of soil pH. They concluded that utilization of subabul stem lignin as a matrix in controlled-release nanoformulation of agrochemicals has multiple advantages, ranging from reduction in harmful effects of herbicides to facilitation of a safer handling system for farmers. More recently, Taverna et al. [47] have developed microspheres of lignin via solvent extraction and evaporation using lignin obtained from organosolv and ionic isolation processes. They compared the efficiency of both types of lignin to encapsulate atrazine through high-performance liquid chromatography, and the release studies were conducted both in water and agriculture soil. The studies demonstrated a higher release profile for organosolv lignin microparticles than for ionic lignin microparticles. Overall, lignin microparticles played a significant role in increasing the dosing time and reducing the leaching of atrazine into the soil as compared with free atrazine. However, the large size of the particles (∼20 μm) may compromise their sticking on the plant surface and blocking of various biochemical processes. Regardless of the antioxidant and antibacterial nature of lignin NPs, their large-scale application for nanoencapsulation/microencapsulation of AIs still faces various challenges related to the toxicity of nonaqueous solvents, the heterogeneous structure of lignin, and the complicated nanofabrication process.

### Cellulose

Cellulose is the most abundant biopolymer obtained from biomass. Unlike lignin, the lack of a variety of functional groups is the major reason for its reduced utilization as an encapsulating agent. There are a few reports published on the use of derivatives of cellulose such as cellulose nanocrystals, nanofibrils, and esters, mostly as comaterials for AI carriers in agriculture [13,48,62]. Milovanovic et al. [48] explored the controlled release profile of CA using thymol as the AI and used supercritical impregnation to prepare solvent-free thymol-impregnated CA beads. They applied the Korsmeyer and Peppas equation (M_t_/M_∞_ = kt^n^, where M_t_ and M_∞_ are the absolute cumulative amounts of thymol released at time t and at infinite release time, respectively, and n and k are the respective release exponent and the kinetic constant) to determine the release kinetics that was dependent on the nature of the release medium and the load of thymol, that is, the higher release of thymol in water only as compared with simulated fluids. The major drawback of using cellulose-based materials as carrier materials is the high affinity of cellulose for water, which results in faster release of the AIs. While nanostructured forms of cellulose are reported to facilitate controlled release of AIs, a certain level of hydrophobicity can be introduced in the carrier molecule via cross-linking [50,62]. Recently, nanofibrillated cellulose extracted from the waste pulp of sugarcane bagasse was used to control the release profile of starch–urea formaldehyde granules [49]. The release of dimethyl phthalate (AI) from the nanofibrillated cellulose–reinforced starch granule barrier was dominated by nanocellulose initially, whereas after the diffusion of water into the granules, it was dominated by water uptake kinetics, that is, a rise in the release of the AI was observed after the swelling of starch in water. To achieve sustained release of polyphenols and control the hydrophilic nature of cellulose, Ciolacu et al. [50] exploited the properties of cellulose and lignin by developing their hybrid hydrogels. They discovered a composition-dependent encapsulation and release profile of the hybrid, that is, a higher lignin content leads to a larger load and release of polyphenol and vice versa (Figure 5d). However, the green approach to incorporate cellulose and lignin together in a matrix had been compromised by the use of epichlorohydrin as the crosslinker. Furthermore, cross-linking of lignin compromises its inherent biodegradability because of the unavailability of various polyphenols after cross-linking.

Although nanoencapsulation is a well-explored field in the controlled and targeted release of drugs, its application for delivery of agrochemicals is still in its infancy. Further studies are required to explain the mode of interaction between the AI and the encapsulating NPs as well as to establish synthesis and encapsulation approaches with tunable release efficiencies in different environmental conditions, including pH, humidity, temperature, and soil chemistry. To develop sustained released media for different types of environments, plants, and AIs, a balance between the AI-surroundings and the carrier–cargo interactions is critical. Recently, Mattos et al. [62] developed a relationship between the thermodynamic parameters of the carrier–cargo (AI)–environment variables. They suggested that the Gibbs free energy (ΔG_r_ = ΔH_r_ + TΔS_r_) should be capable to give an insight into the dissolution of the AI based on the nature of interactions between the AI and the microenvironment (mostly focusing on the solvent) as well as those between the carrier and the AI. The enthalpies (ΔH_r_) and entropies (ΔS_r_) of the system are monitored by cohesive energies within the AI crystal lattice (ΔH_cc_, ΔS_cc_), by interactions between the AI and water (ΔH_aw_, ΔS_aw_) as well as between the AI and the carrier (ΔH_ac_, ΔS_ac_), and also by possible phase change. However, the dissolution of the AI is mainly determined by the enthalpies and entropies contributed by the AI crystal lattice cohesive energies and AI–water interactions because classical dissolution models do not consider the contributions by the carrier [62]. Since negligibly small enthalpy is needed to dissolve water-soluble systems, the dissolution is entropy controlled, and the Gibbs free energy for water-soluble pesticides will be negative, resulting in spontaneous dissolution. On the contrary, to dissolve poorly soluble AI molecules, energy needs to be transferred externally while the respective entropies are negligibly small, resulting in an enthalpy-driven, nonspontaneous reaction (ΔG_r_>0). Based on these relations, it was deduced that the carrier–AI interactions play the dominant role in deciding the release profile of the formulation because the overall free energy of dissolution will be a sum of enthalpic and entropic contributions from the AI cohesive energies and the AI–water, AI–carrier interactions (ΔG_r_ = ΔH_cc_+ΔH_aw_+ΔH_ac_ + T(ΔS_cc_+ ΔS_aw_ +ΔS_ac_). Although this study provides significant insight into the design of the carrier material to tune its release profile, a detailed quantitative understanding of the thermodynamic parameters of the controlled release systems is still a major challenge at this stage.

Although high surface area, small size, and reduced sedimentation of biobased nanoencapsulated formulations make them ideal candidates to be used in sprayables for foliar applications, an in-depth analysis of their wettability and adhesion to leaf surfaces is a largely unexplored area. Developing proper adhesion of the formulation on the plant surface is integral to minimize drift loss and also for controlled release of the AI on various types of surfaces. Owing to the diversity of plant surfaces contributed by variation in chemistry, roughness, shape, and orientation, the spray formulation can be modified to maximize efficacy. Although critical in achieving maximal efficacy, most of the research studies conducted in the field focused on the use of artificial surfaces that cannot completely replicate the real-life situation, for example, formulation droplets mostly do not fall on flat surfaces because the real leaf is mostly sloping downward. Apparent contact angle measurement of a formulation droplet on the leaf surface is an important parameter to determine the wettability and spreading of a formulation on a specific plant. Nairn et al. [77] used a wetting tension dielectric technique to relate the polarity of the leaf surface and various solutions on spreading and adhesion of droplets on leaves. While contact angle (θ) of the droplet can be used to measure spread factor of the liquid, they discovered that the spread factor is also controlled by other variables such as droplet size, critical micelle concentration, surface tension of liquids as well as humidity, surface roughness, and nature of hair present on the leaf surface. Although their study demonstrated reduced spreading of polar solutions on nonpolar leaves and vice versa, no proper protocol was established to minimize the impact of all the interfering real-life factors on the spread and adhesion of the leaf surface. More significantly, their study did not consider the role played by AIs on the status of the droplet on the foliar surface because they used pure solvents or a mixture of solvents (water, acetone, diiodomethane) without any AI. Further understanding of the role played by the interactions between the AI, the carrier, and the microenvironment as well as leaf surface chemistry, roughness, and abundance of leaf hair will lead material scientists and plant pathologists to develop more effective formulations for targeted and controlled delivery of AIs.

## W&P technology

Regardless of the biodegradable nature and ease of availability of the polymers obtained from natural resources, their utilization in agriculture still faces various challenges. Many seed treatment approaches involve complicated processing steps, rendering the product quite expensive for adoption, especially in resource-deficient countries. Unfortunately, people living in those regions are in dire need of better quality and sufficient food production systems, so the need for an economically viable and practical solution is tantamount. The fabrication of biobased substances as fibers or particles in microscale/nanoscale size dimensions also represents an augmented concentration of naturally available substances in an unnatural way, which can ultimately result in ecotoxicity [73]. Therefore, there is a need to establish green technologies for sustainable development of soft materials without the use of nonaqueous solvents and in physical forms that are more compatible with the natural environment.

Among prevalent biomaterials, lignocellulosics hold a promising position because of their abundance and the availability of a variety of functional groups to develop interactions with the loaded cargo. Lignocellulosics are plant-based three-dimensional polymeric composites consisting of cellulose, hemicellulose, and lignin, besides some minor components [51,68,78,79]. While a considerable amount of these materials is produced as agricultural wastes, apart from being used in the pulp and paper industry, most of the wastes are either burned to ash or used as animal feedstock. Recently, some research groups have reported recycling of waste lignocellulose materials or their extracts for various applications, such as sorption [80], packaging [81], feminine hygiene products [82], and composites [83]. As discussed in the previous sections (section Bioplastic–based seed coatings, Encapsulation of pesticides (sub sections on Alginates, and Lignin)), the application of derivatives of lignocellulosic materials for controlled delivery of agrochemicals is also being explored [39,47,50]. However, most of the approaches have focused on various physical forms and components of the parent material to enhance their functionality, which compromises the cost and sustainable nature of this naturally available resource.

**Figure 6:**
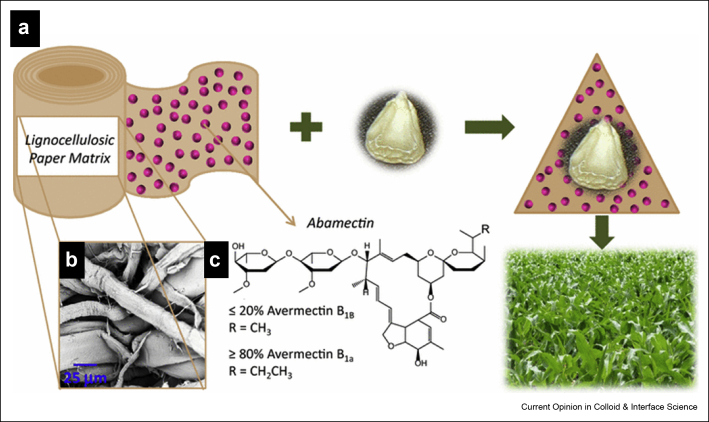
W&P technology in crop protection. **(a)** Schematic of an innovative strategy to use a sustainable lignocellulosic matrix to wrap around the seed/seed pieces and plant for crop protection against plant parasitic nematodes. **(b)** SEM image of the lignocellulosic matrix. The matrix provides a surface-receptive fibrous substrate for the deposition of a crop protection agent such as abamectin . **(c)** Molecular structure of abamectin. Crop protection agent loaded lignocellulosic matrices can be used as seed treatment methodology for smallholder farmers in resource-poor countries where highly technical seed coatings loaded with agrochemicals are not available or financially feasible. The active loaded matrix, which can be manufactured locally, allows them to wrap a seed/seed piece, or even a seedling, for protection against biotic stresses. Reprinted with permission from Cao et al. [51]), Copyright Springer 2016.

In 2016, Cao et al. [51] for the first time reported a novel W&P technology that uses biodegradable matrices developed from various plant fibers (abaca, banana, hardwood, and softwood) for targeted delivery of a biobased anthelmintic Abm. The method entails wrapping (W) matrices impregnated with the AI around seedlings and planting (P) them in the soil, therein giving rise to the label ‘W&P.’ This two-pronged W&P approach presented a sustainable and flexible technology to control soilborne PPNs with the help of a ULV nematicide application. This approach also addresses the issue of limited mobility of Abm in soil by targeting delivery to the precise area of the PPN–plant interaction, resulting in effective control of PPN populations. Although Abm has substantial toxicity to a wide range of nematodes with very low mammalian toxicity, it previously has had limited success in the field because of its propensity for photodegradation and strong binding with soil, making it unavailable soon after it is applied. Banana fiber–based matrices were selected for their slow and sustained release profile, and a W&P approach was introduced for use as wraps for seed pieces (potatoes, yam) at planting (Figure 6). While slow decomposition of the biodegradable banana fiber–based matrix plays a significant role in controlling the release of the loaded cargo, this ‘W&P’ technology is superior to the existing seed coating approaches in its flexibility to produce a structure that is nonsticking as compared with the existing seed treatment approaches. Furthermore, its flexibility to be cut into sheets of variable sizes depending on the sizes of the seed to be wrapped further enhances its scope for user-friendly applications in delivering agrochemicals to a variety of crops.

More recently, the same group [84] has conducted a detailed analysis of the impact of variation in processing conditions on the morphology and sorption/release profile of banana fiber–based matrices to fine-tune the properties of seed wraps. Using the wastes of banana harvests, they used a straightforward, cost-effective, and solvent-free process to develop paper-like lignocellulosic matrices without any chemical additives or crosslinkers. Mechanical beating (refining) of the fiber pulp was the only variable physical condition to fabricate matrices exhibiting fiber morphology and strength different from each other. They conducted greenhouse trials on maize seeds wrapped with matrices to understand the relationship between the strength of the matrices and root penetration profile, that is, to enable the germinating roots to penetrate the wrap without substantial matrix disintegration. These matrices also exhibited a slow and sustained release profile of the AI, when compared with non–banana fiber matrices. While non–banana fiber–based matrices exhibit a lignin content–dependent release profile of the loaded cargo, banana fiber–based matrices also displayed a morphology-dependent release profile of the AI. This trend indicates the unique role played by the fibrillar structure and composition of the banana fiber as well as the fiber processing conditions in dictating the release profile of the matrices. This exciting technology is presently undergoing field trials with yam and potato crops in various parts of sub-Saharan Africa. Initial results demonstrate control of the PPN population and improved crop health and quality of store tubers. (C.H.Opperman et al., vol. 51, pp. 32–33. PO Box 311, Marceline, MO 64658 USA: 58th Society of Nematologists Annual Meeting (SON2019), July 7–11, Raleigh, North Carolina, USA 2019; S.A. Khan et al. 14th IUPAC International Congress of Crop Protection, Paper 5.4.5, Ghent, Belgium, May 2019, T. Pirzada et al. Abstract, B101.3.11, Materials Research Society Fall Meeting, Boston, MI, USA, November 2018).

Among all the existing sustainable crop protection approaches, this innovative W&P technology can be considered a milestone in the development of biodegradable matrices for sustainable crop protection because of its cost-effectiveness, straightforward nature, and flexibility to be applied on various types of seed/seedlings. For tuber crops, the W&P approach represents a major development compared with existing seed treatments by virtue of its flexibility to generate an entity that can be cut into sheets of variable sizes depending on the sizes of the seed piece/seedling to be wrapped. In contrast to existing seed coating and pesticide encapsulation technologies that rely on commercially available precursors and complicated processing, the simplistic and flexible technology in the W&P approach holds promises for a cost-effective biodegradable matrix that can be used for sustained and targeted delivery of agrochemicals, especially for smallholder farmers in resource-deficient areas. Further development of the W&P technology will also begin to address potential applications in more mechanized agricultural settings.

## Conclusions

Broadening applications of agrochemicals has resulted in various issues such as increased leaching of residues into groundwater, evolution of pest resistance, and environmental and human health–related concerns. To minimize the unintended impacts of large-scale applications of synthetic pesticides, there is a dire need of sustainable approaches to develop targeted and controlled ULV delivery platforms that are biodegradable to minimize any harmful impacts on the environment. Herein, we discussed current approaches developed in recent years to protect crops using biodegradable materials for the targeted release of pesticides. In particular, we focused on more traditional film coatings and recently developed nanofibrous coatings, as well as encapsulants of the AI using biopolymers. Although crop protection using biodegradable platforms leads to sustainable agriculture, questions related to the mechanism of reaction and selectivity of matrices for AIs are still unanswered. Furthermore, large-scale application of many of these approaches needs further modification in the fabrication or application process to minimize the costs of the final product for its global application. Although different seed coating techniques and encapsulants using biodegradable materials are playing a major role in the agrochemical sector, the cost and nature of the multistep processes and use of co-chemicals to control the release profile and water solubility of biobased products are the major factors to consider before their large-scale application. Nevertheless, the use of encapsulants is gaining momentum, and considerable effort is being put in that area. More recently, a W&P technology has been introduced to develop biodegradable matrices from the waste of banana harvest via a cost-effective, simplistic, and solvent-free approach. Although these matrices are currently used as seed/seed piece wraps for tuber crops, their ability to be modified for various types of crops and AIs holds promise for the next generation of biodegradable matrices for targeted and controlled delivery of agrochemicals.

## Declaration of competing interest

The authors declare that they have no known competing financial interests or personal relationships that could have appeared to influence the work reported in this paper.
